# Application of preoperative assessment of pain induced by venous cannulation in predicting postoperative pain in patients under laparoscopic nephrectomy: a prospective observational study

**DOI:** 10.1186/s12871-020-01003-z

**Published:** 2020-04-18

**Authors:** Fei Peng, Yanshuang Li, Yanqiu Ai, Jianjun Yang, Yanping Wang

**Affiliations:** Department of Anesthesiology, Pain and Perioperative Medicine, The First Affiliated Hospital of Zhengzhou University, No.1 Jianshe East Road, Zhengzhou, 450052 China

**Keywords:** Venous cannulation, Pain, Postoperative pain, Pain prediction

## Abstract

**Background:**

Postoperative pain is the most prominent concern among surgical patients. It has previously been reported that venous cannulation-induced pain (VCP) can be used to predict postoperative pain after laparoscopic cholecystectomy within 90 mins in the recovery room. Its potential in predicting postoperative pain in patients with patient-controlled intravenous analgesia (PCIA) is worth establishing. The purpose of this prospective observational study was to investigate the application of VCP in predicting postoperative pain in patients with PCIA during the first 24 h after laparoscopic nephrectomy.

**Methods:**

One hundred twenty patients scheduled for laparoscopic nephrectomy were included in this study. A superficial vein on the back of the hand was cannulated with a standard-size peripheral venous catheter (1.1 × 3.2 mm) by a nurse in the preoperative areas. Then the nurse recorded the VAS score associated with this procedure estimated by patients, and dichotomized the patients into low response group (VAS scores < 2.0) or high response group (VAS scores ≥2.0). After general anesthesia and surgery, all the patients received the patient-controlled intravenous analgesia (PCIA) with sufentanil. The VAS scores at rest and on coughing at 2 h, 4 h, 8 h, 12 h, 24 h, the effective number of presses and the number of needed rescue analgesia within 24 h after surgery were recorded.

**Results:**

Peripheral venous cannulation-induced pain score was significantly correlated with postoperative pain intensity at rest (*r*_*s*_ = 0.64) and during coughing (*r*_*s*_ = 0.65), effective times of pressing (*r*_*s*_ = 0.59), additional consumption of sufentanil (*r*_*s*_ = 0.58). Patients with venous cannulation-induced pain intensity ≥2.0 VAS units reported higher levels of postoperative pain intensity at rest (*P* < 0.0005) and during coughing (*P* < 0.0005), needed more effective times of pressing (*P* < 0.0005) and additional consumption of sufentanil (*P* < 0.0005), and also needed more rescue analgesia (*P* = 0.01) during the first 24 h. The odds of risk for moderate or severe postoperative pain (OR 3.5, 95% CI 1.3–9.3) was significantly higher in patients with venous cannulation-induced pain intensity ≥2.0 VAS units compared to those <2.0 VAS units.

**Conclusions:**

Preoperative assessment of pain induced by venous cannulation can be used to predict postoperative pain intensity in patients with PCIA during the first 24 h after laparoscopic nephrectomy.

**Trial registration:**

We registered this study in a Chinese Clinical Trial Registry (ChiCTR) center on July 6 2019 and received the registration number: ChiCTR1900024352.

## Background

Postoperative pain is the most prominent concern among surgical patients. If not adequately controlled, it will affect postoperative rehabilitation, health-related quality of life, and may develop into persistent long-term pain [1–3].

Current postoperative pain management strategies apply a standardized, one-size-fits-all approach to all patients. These standardized protocols are not suitable for the significant difference in patient’s pain and may lead to insufficient analgesia in patients with high analgesic needs, or excessive analgesia, which is accompanied by increasing analgesic-related side effects. The ability to preoperative predict who is at risk for developing moderate or severe postoperative pain might allow anaesthetists to optimize pain management by offering personalized, stratified or targeted analgesic treatment protocols. Preoperative pain prediction methods are highly relevant in this regard.

Numerous studies have tried to identify patients who are at risk for postoperative pain in the preoperative period and evaluated the role of psychological factors and experimental pain tests or quantitative sensory tests (QST) [4–8]. However, none of those prediction methods has been used as a routine for prediction of postoperative pain, mainly because expensive equipment, much time and effort are required outside routine preoperative procedures.

Peripheral venous cannulation, a routine procedure of preoperative preparation, induced pain intensity could be assessed easily and rapidly before surgery without specific equipment or training. It was recently shown that peripheral venous cannulation-induced pain (VCP) intensity could be used to predict the risk of postoperative pain. Patients with VCP score at or above 2.0 VAS units reported higher levels of acute postoperative pain intensity and more often have moderate or severe postoperative pain within 90 mins in the recovery room [9]. Its potential in predicting postoperative pain in patients with patient-controlled intravenous analgesia (PCIA) is worth establishing. The purpose of this study was to test if peripheral VCP intensity can be used to predict the risk for pain in patients with PCIA during the first 24 h after laparoscopic nephrectomy.

## Methods

### Participants

This prospective clinical observational study was approved by the Institutional Scientific Research and Clinical Trials Ethics Committee of the First Affiliated Hospital of Zhengzhou University (ref.: 2019- KY-120) and registered at chictro.org (ref.: ChiCTR1900024352; July 6, 2019).

Patients who were classified as American Society of Anesthesiologists (ASA) physical status I-II, aged between 18 and 65 years, all genders, BMI 18 to 28 kg/m^2^, and scheduled to undergo laparoscopic nephrectomy under general anaesthesia, agree to use postoperative analgesia pump for 48 h after surgery, agreed to cooperate and signed the informed consent were recruited.

Patients with the following conditions were excluded: severe heart, lung and metabolic diseases, hepatic or renal dysfunction, a history of neuromuscular system disease, mental illness, and a tendency to malignant hyperthermia, preoperative existing pain, long-term use of sedative and analgesic drugs (> 3 months), drug or alcohol abusers, or severe hypertension, poor understanding or communication difficulty, failed venous cannulation, changed in surgical approach (from laparoscopic to open surgery, the operative time over than 3 h, failed to complete the data collection.

### Preoperative pain assessment

An anaesthetist visited the patients the day before surgery, described the visual analogue scale (VAS) for them and instructed on the use of PCIA bump. On the day of surgery, an experienced nurse inserted a peripheral venous catheter (B. Braun Melsungen AG, Germany) with a standard-size (1.1 × 3.2 mm inner diameter) into a superficial vein on the back of the patient’s hand in the preoperative preparation room. The patients were asked to estimate, on a horizontal VAS ruler, their maximum pain intensity associated with this procedure, recorded to one decimal point (0.0–10.0). Then the nurse recorded the VAS score estimated (on a horizontal VAS ruler, their maximum pain score associated with this procedure) by the patients, and dichotomized the patients into low response group (VCP score < 2.0 VAS units) or high response group (VCP score ≥ 2.0 VAS units). The nurse was aware of whether the patient was to take part in the study.

### Anaesthesia

All patients were anaesthetized by anaesthetists who were blinded to study and did not participate in data collection. Once in the operating room, the standardized monitoring of ECG, SpO_2_, noninvasive blood pressure was established. Before the induction of anaesthesia, the patients were given IV Penehyclidine Hydrochloride 0.5 mg, and a loading dose of dexmedetomidine with 0.5 μg kg^− 1^ was infused over 10 min. The bispectral index (BIS) was used to monitor the depth of anaesthesia. Then anaesthesia was induced with midazolam 2 mg, sulfentanil 0.5 μg kg^− 1^, etomidate 2–3 mg kg^− 1^. Cisatracurium 0.2 mg kg^− 1^ was given to facilitate endotracheal intubation. Anaesthesia was maintained with sevoflurane (1–2%), remifentanil 0.1–0.3 μg kg^− 1^ min^− 1^. The cisatracurium was used to provide a satisfactory level of muscle relaxation. The BIS value was maintained between 40 to 60. The pneumoperitoneum pressure with carbon dioxide was set at 13–15 mmHg, and the EtCO_2_ was maintained at 35 to 45 mmHg. Thirty minutes before the end of the surgery, sulfentanil 10 μg and flurbiprofen axetil 100 mg were given as postoperative analgesia, and tropisetron 5 mg was given to prevent postoperative nausea and vomiting. At the end of the surgery, sevoflurane and remifentanil were stopped. Immediately after surgery, the PCIA pump was attached to the peripheral venous line by the anaesthetist. Then the patients were sent to the post anaesthetic care unit for anaesthetic resuscitation. All patients were sent to the general ward after being fully awake. Upon arrival in the general ward, all the patients were once again instructed on the use of the PCIA pump and VAS.

### Postoperative analgesia regimen

The PCIA with sulfentanil regimen was applied to 48 h after surgery. The PCIA regimen consisted of sulfentanil 3.0 μg kg^− 1^ and 5 mg tropisetron, mixed with 0.9% normal saline to a total volume of 150 ml. The PCIA was programmed to deliver a 2 ml bolus on demand, with a lock-out interval of 10 min, and a background infusion rate of 2 ml h^− 1^. In the ward, patients pressed PCA when VAS score at rest > 3.0. If patients still reported pain or the VAS scores ≥4.0, supplemental rescue boluses of intravenous flurbiprofen axetil injection of 50 mg were administered. The complete history of continuous infusion, bolus infusion, and bolus demand for the PCIA device was downloaded after surgery.

### Outcome variables measures and data collection

The study outcomes variables and the vital parameters were recorded at 2, 4, 8, 12 and 24 h after surgery. During the studied period, pain intensity, sulfentanil consumption, pressing times of the PCIA, and the number of rescue analgesia were recorded at above time points. Overall satisfaction index of the patients was recorded at 24 h. Pain intensity was assessed with VAS at rest and during coughing.

The primary outcome was maximum postoperative pain scores at rest and during coughing within the first 24 h. The secondary outcome was effective times of pressing, additional consumption of sulfentanil and satisfaction index at 24 h. Also, the number of rescue analgesia within the first 24 h was also measured.

Postoperative data collector was blinded to the preoperative peripheral venous cannulation-induced pain score of the patients.

### Sample size and statistical analyses

The sample size was based on a pilot experiment of 20 cases resulted in our observation that patients with VCP score ≥ 2.0 VAS units was present in 8 out of 20 patients and that mean maximum postoperative pain score (VAS) at rest within 24 h after surgery was 3.9 (± 1.3). Therefore, in order to show a 20% difference between the patients with VCP score ≥ 2.0 VAS units and the patients with VCP score ≥ 2.0 VAS units, the number of patients in each group was expected to be 41 (α = 0.05, β = 0.8). Since the groups are unequal, assuming a 40% of patients with VCP score ≥ 2.0 VAS units, and allow for up to 15% dropouts, a total of 120 patients (48 patients with VCP score ≥ 2.0 VAS units and 72 patients with VCP score < 2.0 VAS units) would be sufficient to test our hypothesis.

The IBM SPSS version 22.0 software packages were used for statistical analyses. The normality of the continuous data was tested by the Shapiro-Wilk test. Normally distributed continuous variables were expressed as mean ± SD and compared between groups using a two-sample Student *t*-test. IF the distribution was not normal, the median with inter-quartile range (IQR) were expressed, and a Mann–Whitney *U*-test was used. Categorical data were expressed as frequency (n) and percentage (%) and were statistically tested using the chi-square or Fisher’s exact test. Correlations between variables were assessed with Spearman’s rank correlation coefficients. Logistic regression analysis was used to evaluate the predictive abilities of cannulation-induced pain intensity. All *P* values <0.05 were considered to be statistically significant.

## Results

**Fig. 1:**
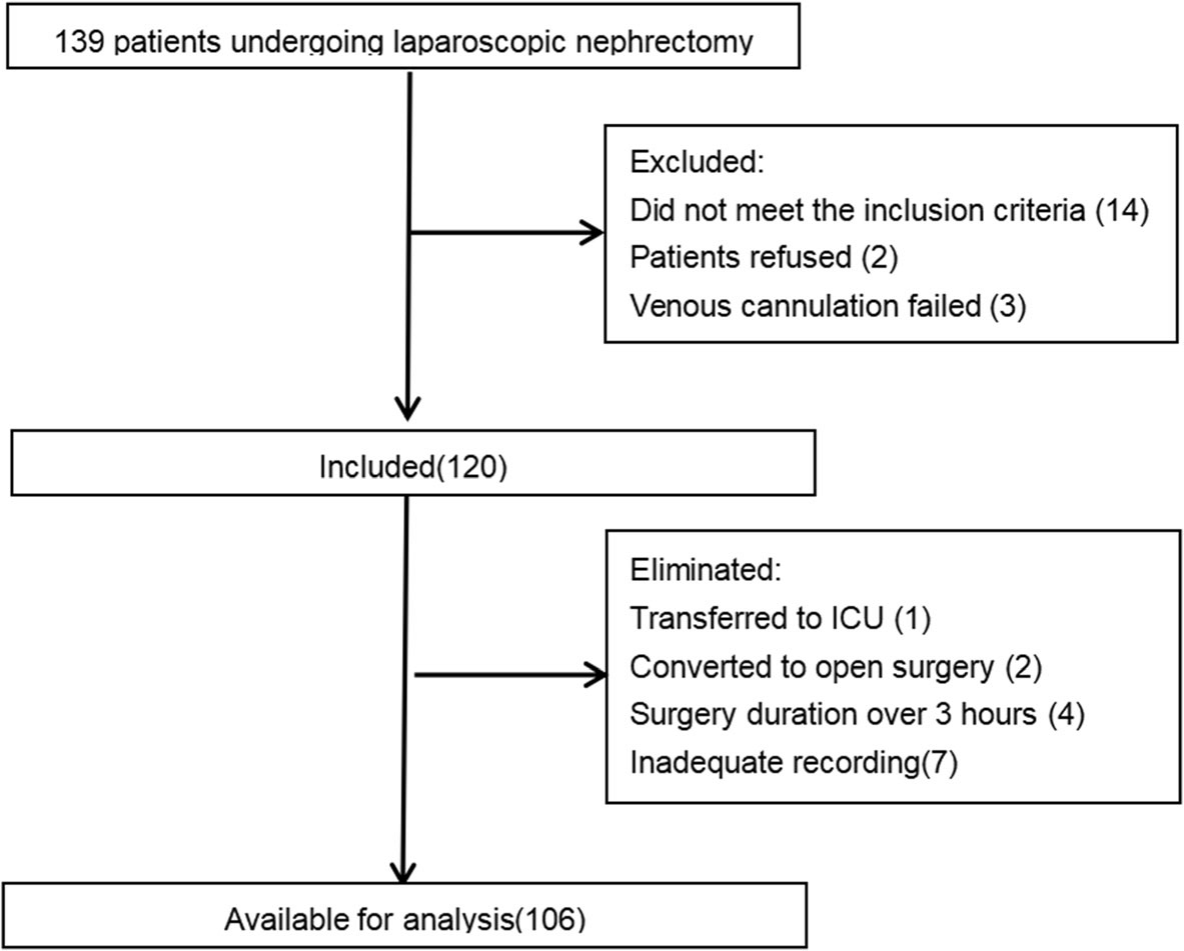
Enrollment flow chart of patients

A total of 139 patients undergoing laparoscopic nephrectomy were screened between August and October 2019, of which 19 were excluded because they did not meet the inclusion criteria, refused to participate in the trial, and failed venous cannulation. Among the remaining 120 patients, some were eliminated due to transferred to ICU, converted to open surgery, surgery duration over 3 hours, or incomplete recording, and 106 study patients were available for analysis (Fig. 1).

Patients’ demographic characteristics and perioperative data are shown in Table 1. The median (inter-quartile range) of peripheral venous cannulation-induced pain score, postoperative maximum pain score at rest, postoperative maximum pain score during coughing, effective times of pressing, additional consumption of sulfentanil were 1.8 (1.4–2.6), 3.4 (3.0–3.9), 5.8 (5.5–6.3), 1 (0–5), 3.18 (0–12.56) μg. The cut-off point of classification according to VCP pain score (2.0 VAS units) was close to the median level of peripheral venous-induced pain score.

**Table 1:** Patients’ demographic characteristics and perioperative data

Variable	
Age (years)	53 (40–59)
Gender (M/F)	57/49
ASA (I/II)	38/68
BMI (kg/m^2^)	24.8 (22.1–26.5)
Peripheral venous cannulation-induced pain score	1.8 (1.4–2.6)
Postoperative maximum pain score at rest	3.4 (3.0–3.9)
Postoperative maximum pain score during coughing	5.8 (5.5–6.3)
Effective times of pressing	1 (0–5)
Additional consumption of sulfentanil, μg	3.18 (0–12.56)

Data are presented as median (range) or number

**Fig. 2:**
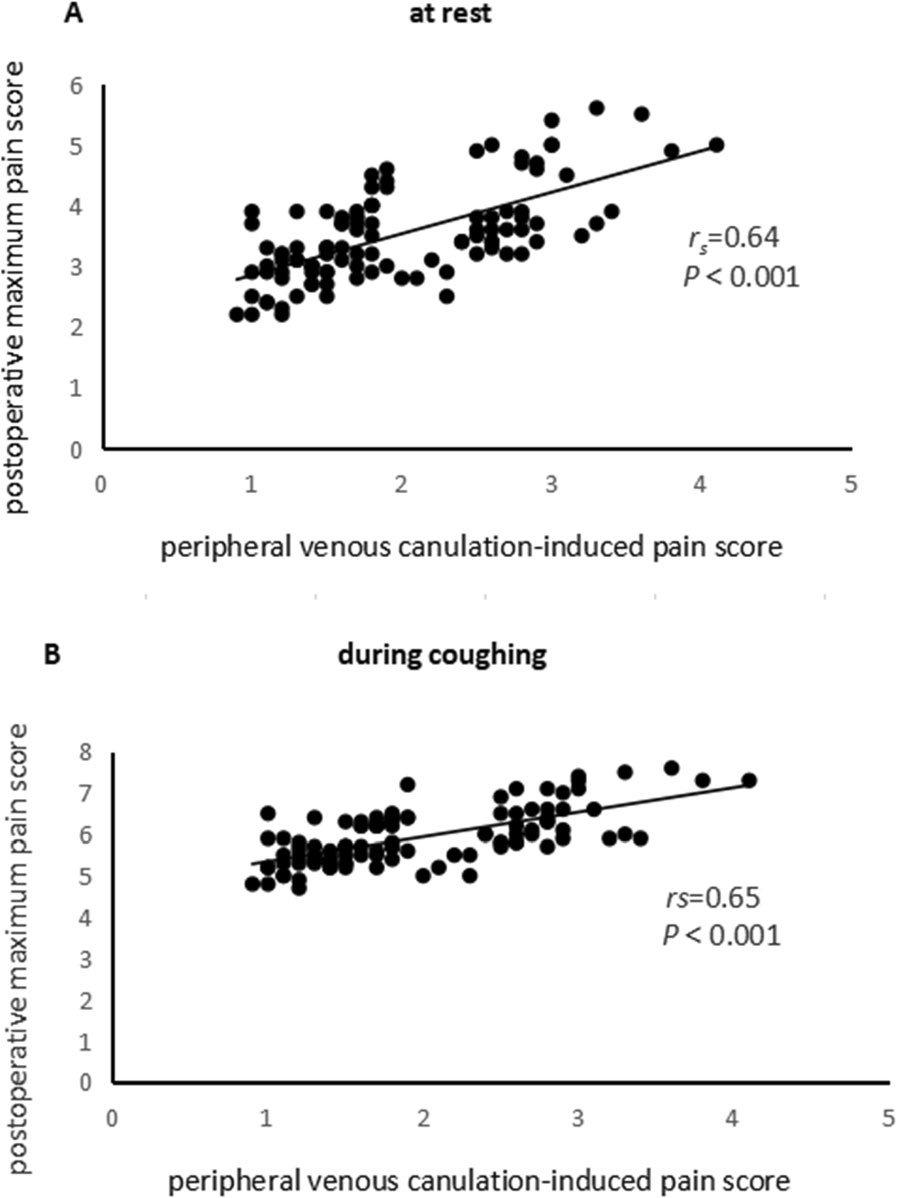
Scatter plot of peripheral venous cannulation-induced pain score and postoperative maximum pain score (**a**) at rest, (**b**) during coughing

Bivariate correlations between peripheral venous cannulation-induced pain score and outcome variables are shown in Fig. 2 and Table 2. Postoperative maximum pain score at rest (*r*_*s*_ = 0.64, *P* < 0.001) (Fig. 2a), postoperative maximum pain score during coughing (*r*_*s*_ = 0.65, *P* < 0.001) (Fig. 2b), effective times of pressing (*r*_*s*_ = 0.59, *P* < 0.001), additional consumption of sulfentanil (*r*_*s*_ = 0.58, *P* < 0.001) were statistically significantly correlated with peripheral venous cannulation-induced pain score, respectively.

**Table 2:** Bivariate correlations between venous cannulation-induced pain score and outcome variables

	Correlation coefficient (*r*_*s*_)
Postoperative maximum pain score at rest	0.64^*^
Postoperative maximum pain score during coughing	0.65^*^
Effective times of pressing	0.59^*^
Additional consumption of sulfentanil, μg	0.58^*^

^*^meant *P* < 0.05

Patients’ demographic characteristics and perioperative data between the two groups are shown in Table 3. There were no significant differences in age, gender, BMI, ASA, history of surgery, type of surgery, approaches of surgery, duration of anaesthesia and surgery, consumption of remifentanil between the two groups. Patients with venous cannulation-induced pain intensity ≥2.0 VAS units reported higher levels of postoperative pain intensity at rest (3.7 vs. 3.2 VAS units; *P* < 0.0005) and during coughing (6.2 vs. 5.6 VAS units; *P* < 0.0005), needed more effective times of pressing (3 vs. 1; *P* < 0.0005) and additional consumption of sulfentanil (7.46 vs. 2.56 μg; *P* < 0.0005), and also needed more rescue analgesia (33.3% vs. 12.5%; *P* = 0.01) during the first 24 h. While the satisfaction index was significantly lower (3 vs.5; *P* < 0.0005).

**Table 3:** Demographic characteristics and perioperative data in patients dichotomized for peripheral venous cannulation-induced pain score

	venous cannulation-induced pain score (VAS units)		
	<2.0	≥ 2.0	*P* values
Total number of patients	64	42	
Gender (M/F)	39/25	18/24	0.068
Age (years)	53 (48–62)	52 (38–58)	0.248
BMI (kg/m^2^)	24.2 (22.1–25.9)	25.1 (22.1–27.8)	0.076
ASA (I/II)	23/41	15 /27	0.981
History of surgery (No/Yes)	40 /24	22/20	0.301
Type of surgery (Partial/Radical)	24/40	13/29	0.489
Approaches of surgery (Retroperitoneal/Transperitoneal)	15/49	9/33	0.809
Duration of anaesthesia, min	134 (100–157)	133 (113–170)	0.339
Refentanil, mg	1.05 (0.80–1.40)	1.10 (1.00–1.36)	0.361
Postoperative maximum pain score at rest	3.2 (2.9–3.7)	3.7 (3.4–4.7)	< 0.0005
Postoperative maximum pain score on coughing	5.6 (5.3–6.2)	6.2 (5.8–7.0)	< 0.0005
Additional consumption of sulfentanil, μg	2.56 (0–7.86)	7.46 (2.71–22.91)	< 0.0005
Effective times of pressing	1 (0–3)	3 (1–8)	< 0.0005
Needed rescue analgesia	8 (12.5%)	14 (33.3%)	0.01
satisfaction index	5 (4–5)	3 (3–4)	< 0.0005

Variables are presented as median (range) or number

The number of patients experiencing maximum pain (at rest) exceeding 4.0 VAS units during the first 24 h between high response group and low response group were shown in Table 4. In high response group, 33.3% reported moderate or severe postoperative pain. While, in low response group, 12.5% reported moderate or severe pain. There was statistically significant in the risk for moderate or severe pain between two groups (*P* = 0.01).

**Table 4:** Cross-tabulation for a prediction model for maximum postoperative pain according to peripheral venous cannulation-induced pain score

	Venous cannulation-induced pain score (VAS units)		Total number of patients
	< 2.0	≥ 2.0	
Patients reporting maximum postoperative pain intensity at rest (VAS units)			
< 4	56 (87.5%)	28 (66.7%)	84
≥ 4	8 (12.5%)	14 (33.3%)	22
Total number of patients	64	42	106

Comparison of the number of patients experiencing pain exceeding VAS 4.0 within 24 h between high response group and low response group (*P* = 0.01)

After controlling for possible factors affecting postoperative pain (gender, age, history of surgery, type of surgery and approaches of surgery), the odds of risk for moderate or severe postoperative pain (OR 3.5, 95% CI 1.3–9.3) was significantly higher in patients with venous cannulation-induced pain intensity ≥2.0 VAS units compared to those < 2.0 VAS units (Table 5).

**Table 5:** Logistic regression analysis of the ability of venous cannulation-induced pain score (≥ / < 2.0 VAS units) to predict postoperative pain intensity ≥4.0 VAS units

	multivariate analysis	
	OR(95% CI)	*P* value
Venous cannulation-induced pain score (VAS units)		
< 2.0	1.0(ref)	0.012
≥ 2.0	3.5 (1.3–9.3)	

Abbreviations = OR (odds ratio), CI (confifidence interval)

The model adjusted for gender, age, history of surgery, type of surgery and approaches of surgery

## Discussion

This study shows that peripheral venous cannulation-induced pain score is positively correlated with postoperative maximum pain and addition consumption of sulfentanil during the first 24 h after laparoscopic nephrectomy. Patients with VCP score ≥ 2.0 VAS units are more likely to report higher postoperative pain scores and additional consumption of sulfentanil. Furthermore, patients with VCP score ≥ 2.0 VAS units have a 3.5 times higher risk for moderate or severe postoperative pain.

Peripheral venous cannulation, is a routine practice before surgery, has been reported by patients to be painful. The pain intensity is associated with cannula site, cannula size, failed venipuncture attempts and process times [10, 11]. In the present study, we did not include patients with failed venipuncture, and this procedure was performed by the experienced nurse with same diameter puncture needle on the same site. The results showed that the median level of pain intensity associated with venous cannulation on the hand was 1.8 VAS units, which was close to current study proposed the cut-off level of the VCP (2.0 VAS units). Furthermore, the cut-off level of the VCP was found to be 2.0 VAS units in predicting postoperative pain in Persson et al. Study [9]. Another study reported that 2.0 VAS units represented a more reasonable cut-off level of VCP for prediction of postoperative pain [12].

Prior researchers have also reported similar results to ours. Persson et al. [9] study reported that the VCP score was significantly correlated with postoperative maximum pain score at rest within 90 mins after laparoscopic cholecystectomy. Patients with VCP score ≥ 2.0 VAS units had higher postoperative pain and risk for moderate or severe postoperative pain in the recovery room. However, the proportion of moderate or severe postoperative pain in patients with VCP score ≥ 2.0 VAS units and < 2.0 VAS units were higher than the present study reported. In our study, 33.3 and 12.5% of patients reported moderate or severe postoperative pain between patients with VCP score ≥ 2.0 VAS units and < 2.0 VAS units, while in Persson et al. [9] study, the proportions were 77 and 35%. A possible explanation as to the difference is that patients are administered opioid through PCIA with continuous background infusion instead of on-demand. Carvalho et al. [13] study used VCP score to predict labor pain of 50 women and found that pain intensity of intravenous cannulation was correlated with time to epidural request during the course of induction of labour. A recently study [12] evaluated the usefulness of VCP score in 4 categories surgery (presumed to with hardly any postoperative pain, slight, moderate and severe levels of postoperative pain) and reported that the method of VCP score was only statistically significant in the patients subjected to surgery presumed to result in moderate levels of postoperative pain.

Numerous studies have been developed to predict postoperative pain in the last decade. The pain intensity of QST stimuli, including pressure, electric, and thermal stimulus, have been reported to correlate with the intensity of postoperative pain [6–8, 14–17]. Werner et al. [18] reported that QST assessments might predict up to 54% of the variance in the postoperative pain experience. The predictive ability of the tests is much higher than previously reported for single-factor analyses of demographics and psychologic factors. However, these methods may be time-consuming and not necessarily available in a fast-paced clinical environment. The VCP assessment is easy to perform, timely and rapid clinical test and requires no special equipment.

Our finding has important clinical implications since pain management in the postoperative period still present a challenge. There is large individual variability in response to opioids and the potential side effects such as respiratory depression, nausea, vomiting and constipation. The ability to preoperative identification of patients at greater risk for moderate or severe postoperative pain and having higher opioids requirements is very beneficial. Using peripheral VCP intensity as a predictor of postoperative pain may allow anaesthetists more convenient to adjust the dosage of opioids and can potentially improve postoperative pain management.

There are some limitations to the present study. Known risk factors of postoperative pain are female gender, lower age, preoperative pain, intraoperative factors and psychological factors, and so on [8, 19–21]. Furthermore, it was reported that psychological factors were correlated with venous cannulation-induced pain score [22]. This study did not take psychological factors into account. The psychological variables may affect the levels of postoperative pain and venous cannulation-induced pain score. Besides, this method did not be evaluated in other surgical patients.

## Conclusions

In conclusion, peripheral venous cannulation-induced pain score was associated with postoperative pain and addition consumption of sulfentanil during the first 24 h after laparoscopic nephrectomy. Patients with VCP score ≥ 2.0 VAS units had higher postoperative pain scores, additional consumption of sulfentanil and risk for moderate or severe postoperative pain. Therefore, peripheral venous cannulation-induced pain intensity can be considered as a simple and useful method to predict postoperative pain in patients with PCIA during the first 24 h after laparoscopic nephrectomy.

## Data Availability

The datasets generated and analysed during the current study are available from the corresponding author on reasonable request.

## Abbreviations

VCP: Venous cannulation-induced pain
PCIA: Patient-controlled intravenous analgesia
VAS: Visual analogue scale
QST: Quantitative sensory tests
ASA: American Society of Anesthesiologists
BMI: Body mass index
